# Karyotype heterogeneity in *Philodendron* s.l. (Araceae) revealed by chromosome mapping of rDNA loci

**DOI:** 10.1371/journal.pone.0207318

**Published:** 2018-11-15

**Authors:** Emanuelle Varão Vasconcelos, Santelmo Vasconcelos, Tiago Ribeiro, Ana Maria Benko-Iseppon, Ana Christina Brasileiro-Vidal

**Affiliations:** 1 Departamento de Genética, Centro de Biociências, Universidade Federal de Pernambuco, CEP, Recife, Pernambuco, Brazil; 2 Instituto Tecnológico Vale, CEP, Belém, Pará, Brazil; 3 Departamento de Botânica, Centro de Biociências, Universidade Federal de Pernambuco, CEP, Recife, Pernambuco, Brazil; 4 Instituto Federal de Mato Grosso, Campus Avançado de Diamantino, Rodovia Roberto Campos, CEP, Diamantino, Mato Grosso, Brazil; Tulane University Health Sciences Center, UNITED STATES

## Abstract

*Philodendron* s.l. (Araceae) has been recently focus of taxonomic and phylogenetic studies, but karyotypic data are limited to chromosome numbers and a few published genome sizes. In this work, karyotypes of 34 species of *Philodendron* s.l. (29 species of *Philodendron* and five of *Thaumatophyllum*), ranging from 2*n* = 28 to 36 chromosomes, were analyzed by fluorescence *in situ* hybridization (FISH) with rDNA and telomeric probes, aiming to understand the evolution of the karyotype diversity of the group. *Philodendron* presented a high number variation of 35S rDNA, ranging from two to 16 sites, which were mostly in the terminal region of the short arms, with nine species presenting heteromorphisms. In the case of *Thaumatophyllum* species, we observed a considerably lower variation, which ranged from two to four terminal sites. The distribution of the 5S rDNA clusters was more conserved, with two sites for most species, being preferably located interstitially in the long chromosome arms. For the telomeric probe, while exclusively terminal sites were observed for *P*. *giganteum* (2*n* = 30) chromosomes, *P*. *callosum* (2*n* = 28) presented an interstitial distribution associated with satellite DNA. rDNA sites of the analyzed species of *Philodendron* s.l. species were randomly distributed considering the phylogenetic context, probably due to rapid evolution and great diversity of these genomes. The observed heteromorphisms suggest the accumulation of repetitive DNA in the genomes of some species and the occurrence of chromosomal rearrangements along the karyotype evolution of the group.

## Introduction

Araceae (ca. 3600 species) is a widely distributed monocot family with high ecological diversity, occurring mainly in tropical regions [1, 2]. *Philodendron* s.l. is the second largest group within the family (ca. 500 species), presenting a wide Neotropical distribution, with the Amazon region as its probable center of origin [2–4]. The genus was traditionally divided into three monophyletic subgenera: *P*. subg. *Meconostigma* (now recognized as the genus *Thaumatophyllum*, with 21 species), mostly with the diploid number 2*n* = 36; *P*. subg. *Pteromischum* (82 species), with chromosome counts only for two species (both with 2*n* = 32); and *P*. subg. *Philodendron* (ca. 400 species), as the most morphologically diverse group, with chromosome number ranging from 2*n* = 26 to 40 chromosomes, although 2*n* = 32 and 34 have been reported as the most common numbers [5–10]. It is suggested that *x* = 16 is the basic number for *Philodendron* s.l. (including *Thaumatophyllum*) and that the karyotype evolution of the group has been driven by both ascending and descending dysploidy events, since no polyploidy was reported so far [8, 9].

The ribosomal RNA (rRNA) genes have been physically mapped in the chromosome of species of several plant groups [11–13], being useful for understanding the general patterns of karyotype evolution among related species and for cytotaxonomic approaches [14–16]. In Araceae, studies involving the physical location of 5S and 35S rDNA by FISH (Fluorescent *In Situ* Hybridization) are available for just a few species, including 10 species of *Typhonium* [17] and 17 other species from different groups, such as *P*. *hederaceum* (Jacq.) Schott (as *P*. *scandens* Koch & Sello), two species of *Anthurium*, three species of *Spathiphyllum* and two species of *Ulearum* [18, 19]. Overall, all analyzed species presented only one pair of 5S rDNA sites, located in the subterminal or interstitial regions, whereas for 35S rDNA there was a predominance of four terminal sites, with few exceptions.

Thus, based on the chromosome number and genome size variation previously mentioned for *Philodendron* and *Thaumatophyllum* [8, 9, 20], we have addressed the following questions: (I) Is the distribution of rDNA sites in *Philodendron* and the sister genus *Thaumatophyllum* conserved? (II) Does the distribution pattern of rDNA sites agree with the available phylogenetic data? To answer these questions, molecular cytogenetic data were generated for the first time for 29 *Philodendron* and five *Thaumatophyllum* species by FISH with rDNA and telomeric probes. In addition, these data were plotted in a recently published phylogenetic tree of the group [21], aiming to better understand the patterns of karyotype evolution of both genera.

## Materials and methods

### Plant material and chromosomal preparations

Thirty-four species were analyzed by FISH. Sixteen species were sampled from the living collection kept at the Royal Botanic Gardens, Kew (Richmond, United Kingdom), and 18 were collected in different regions of Brazil, as licensed by the Brazilian authorities (ICMBio license numbers 14311–2 and 31038–4). The collected Brazilian plants are being cultivated in the living collection of the Laboratory of Plant Genetics and Biotechnology, Department of Genetics, UFPE (Recife, Brazil). The provenance of the plants and accession numbers are shown in Table 1.

**Table 1:** Species of *Philodendron* and *Thaumatophyllum* with their respective section and accession, chromosome complement size, chromosome range size, diploid number and number of rDNA sites.

Genus	Section	Species	Provenance and accession number	2*n*	Number of rDNA sites	
					35S	5S
***Thaumatophyllum***		*T*. *corcovadense* (Kunth) Sakur., Calazans & Mayo	Taquaritinga do Norte, Pernambuco, Brazil; Cultivated at LGBV; SV314	36	4	2
		*T*. *lundii* (Warm.) Sakur., Calazans & Mayo	Morro do Chapéu, Bahia, Brazil; cultivated at LGBV; SV089	36	2	2
		*T*. *mello-barretoanum* (Burle-Marx ex G.M. Barroso) Sakur., Calazans & Mayo	Recife, Pernambuco, Brazil; Cultivated at LGBV; SV534	34	2	2
		*T*. *saxicola* (Krause) Sakur., Calazans & Mayo	Mucugê, Bahia, Brazil; Cultivated at LGBV; SV539	36	2	2
		*T*. *spruceanum* Schott	Reserva Florestal Adolpho Ducke, Amazonas, Brazil; Cultivated at LGBV; SV063	32	4	2
***Philodendron*** **subg. *Philodendron***	*Baursia*	*P*. *callosum* K.Krause	Presidente Figueiredo, Amazonas, Brazil; Cultivated at LGBV; SV022	28	-	-
		*P*. *glaziovii* Hook.f.	Pedra Azul, Espírito Santo, Brazil; Cultivated at RBG Kew/ 1983–2011	34	6	2
		*P*. *renauxii* Reitz	Itapema, Santa Catarina, Brazil; Cultivated at RBG Kew/ 1983–1988	34	8	2
	*Macrobelium*	*P*. *annulatum* Croat	Cerro Jefe, Panamá; Cultivated at RBG Kew/ 1996–4421	32	2	2
		*P*. *barrosoanum* G.S.Bunting	Reserva Florestal Adolpho Ducke, Amazonas, Brazil; Cultivated at LGBV; MC107	32	8	2
		*P*. *burle-marxii* G.M.Barroso	Amazonas region, Colombia; Cultivated at RGB Kew/ 1975–98	34	10	2
		*P*. *eximium* Schott	Taquaritinga do Norte, Pernambuco, Brazil; Cultivated at LGBV; SV293	32	6	2
		*P*. *inconcinnum* Schott	Cultivated at RBG Kew/ 1981–3728	32	2	2
		*P*. *krugii* Engl.	Trinidad and Tobago; Cultivated at RBG Kew/ 1980–1645	34	2	2
		*P*. *quinquenervium* Schott	Uatumã, Amazonas, Brazil; Cultivated at LGBV; SV076	32	15	2
		*P*. *smithii* Engl.	Tabasco, Mexico; Cultivated at RBG Kew/ 1980–1583	32	8	2
		*P*. *uleanum* Engl.	Napo, Ecuador; Cultivated at RGB Kew/ 1982–1568	34	12	2
	*Philodendron*	*P*. *billietiae* Croat	Cultivated at RBG Kew/ 2005–2363	32	16	2
		*P*. *fragrantissimum* (Hook.) G.Don	Igarassu, Pernambuco, Brazil; Cultivated at LGBV; SV295	32	2	2
		*P*. *giganteum* Schott	Oriole trail, Montserrat; Cultivated at RBG Kew/ 2011–1735	30	10	2
		*P*. *hederaceum* (Jacq.) Schott	Floresta da Tijuca, Rio de Janeiro, Brazil; Cultivated at LGBV; SV248	32	2	2
		*P*. *maximum K*.*Krause*	Cultivated at RBG Kew/ 1973–381	34	12	2
		*P*. *megalophyllum* Schott	Uatumã, Amazonas, Brazil; Cultivated at LGBV; SV320	34	12	2
		*P*. *melinonii* Brongn.ex Regel	Reserva Florestal Adolpho Ducke, Amazonas, Brazil; Cultivated at LGBV; MC085	30	4	2
		*P*. *schmidtiae* Croat & C.E.Ceron	Napo, Ecuador; Cultivated at RBG Kew/ 1982–1573	32	14	2
		*P*. *tenue* K.Koch & Augustin	Costa Rica; Cultivated at RGB Kew/ 1984–612	34	6	2
	*Polytomium*	*P*. *distantilobum* K.Krause	Uatumã, Amazonas, Brazil; Cultivated at LGBV; SV318	32	2	2
		*P*. *lacerum* (Jacq.) Schott	Guiana; Cultivated at RBG Kew/ 1979–3173	32	10	2
	*Schizophyllum*	*P*. *bipennifolium* Schott	INPA, Acre, Brazil; Cultivated at LGBV; SV307	32	8	3
		*P*. *nadruzianum* Sakur.	Floresta da Tijuca, Rio de Janeiro, Brazil; Cultivated at LGBV; LSBC175	32	2	2
		*P*. *pedatum* (Hook.) Kunth	Floresta da Tijuca, Rio de Janeiro, Brazil; Cultivated at LGBV; MC081	32	4	2
		*P*. *quinquelobum* K.Krause	Urucu, Amazonas, Brazil; Cultivated at LGBV; MC080	32	4	2
	*Tritomophyllum*	*P*. *angustilobum* Croat & Grayum	Heredia, Costa Rica; Cultivated at RBG Kew/ 1996–4420	34	3	2
		*P*. *tripartitum* (Jacq.) Schott	Chiapas, México; Cultivated at RBG Kew/ 1980–1524	34	6	2

Abbreviations: LGBV: Laboratory of Genetics and Plant Biotechnology, Federal University of Pernambuco (Recife, Brazil); RBG Kew: Royal Botanic Gardens, Kew (Richmond, United Kingdom).

Root tips were pretreated in 2 mM 8-hydroxyquinoline at 8°C for 24 h, fixed in Carnoy solution (ethanol:acetic acid, 3:1, v/v) at room temperature for 24 h, and stored at -20°C. Subsequently, the fixed root tips were washed in distilled water and digested in an enzyme solution containing 2% cellulase (w/v) (Onozuka R-10, Serva) and 20% pectinase (v/v) (Sigma-Aldrich) overnight at 37°C. Slides were prepared by squashing the meristematic tissue in 45% acetic acid. In addition, slides were stained in a solution of 2 μg mL^-1^ DAPI (4', 6-diamidino-2-phenylindole) and glycerol (1:1, v/v) and then analyzed. Afterwards, the best slides were de-stained and fixed in Carnoy solution for 30 min and transferred to absolute ethanol for 1 h, both at room temperature. After air drying, the slides were stored at -20°C. At least five root tips were analyzed per species.

### Probes, fluorescent *in situ* hybridization and data analysis

The rDNA probes used for FISH were R2, a 6.5 kb fragment containing the 18S-5.8S-25S rDNA unit from *Arabidopsis thaliana* (L.) Heynh., and D2, a 400 bp fragment containing two 5S rDNA units from *Lotus japonicus* (Regel) K.Larsen [22]. Labelling was performed by nick translation with digoxigenin-11-dUTP (Roche Diagnostics) and biotin-11-dUTP (Roche Diagnostics) for 35S and 5S rDNA, respectively. The telomeric probe was amplified by PCR according to Ijdo et al. [23], with the primers (TTTAGGG)_5_ and (CCCTAAA)_5_ and labeled with Cy3-dUTP (Jena Bioscience) as described before.

The FISH procedures followed Vasconcelos et al. [24], except for the chromosome denaturing, which occurred separately from the probe in 70% formamide in 2×SSC at 80–85°C for 7 min, and then dehydrated for 5 min in an alcoholic series (ethanol 70% and 100%) at -20°C. The stringency wash was performed in 0.1×SSC at 42°C. The hybridization mix consisted of 50% (v/v) formamide, 10% (w/v) dextran sulfate, 2×SSC and 2–5 ng/μL of probe. Digoxigenin and biotin-labeled probes were detected using conjugated anti-digoxigenin rhodamine (Roche), and Alexa Fluor conjugated streptavidin (Invitrogen), respectively, in 1% (w/v) BSA. Preparations were counterstained and mounted with 2 μg/mL DAPI in Vector’s Vectashield (1:1; v/v).

Images were captured using a Leica DMLB epifluorescence microscope coupled with a Leica DFC 340FX camera, using the Leica CW 4000 software. Images were optimized for better brightness and contrast with Adobe Photoshop CS3 (Adobe Systems Incorporated). The 35S rDNA was pseudocolored in green, and the 5S rDNA was pseudocolored in red. At least eight images were analyzed per species.

Also, for a better understanding of the karyotype evolution of the genus, idiograms of the chromosomes carrying rDNA sites of each species were plotted in the phylogenetic tree previously reported for the group [21]. Thus, chromosome lengths of three metaphases for each species with similar condensation pattern were measured twice considering both chromatids, totalizing six measurements per species, using the MicroMeasure v3.3 software [25]. The Adobe Flash CS3 program (Adobe Systems Incorporated) was used for the elaboration of the idiograms.

### Genomic DNA extraction, sequencing and satellite DNA analysis

Genomic DNA of *P*. *callosum* K.Krause was extracted from fresh leaves using the CTAB protocol described by Weising et al. [26]. The precipitation of contaminating polysaccharides was performed according to Michaels et al. [27]. Genomic DNA was sequenced in Illumina MiSeq (2 × 250 pb). Clusterization and characterization of the repetitive genome fraction were performed on the *Galaxy/RepeatExplorer* platform using the Elixir-cerit server. The TAREAN tool was used to identify satellite DNA sequences (https://repeatexplorer-elixir.cerit-sc.cz) [28–30]. Cluster sharing similarity to the telomeric motif was manually checked and the contigs were used to reconstruct the monomer.

## Results

Chromosome number ranged from 2*n* = 28 to 36 considering the total of 34 analyzed species (Table 1): 2*n* = 28 (*P*. *callosum*); 2*n* = 30 (*P*. *giganteum* and *P*. *melinonii*), 2*n* = 32 (*T*. *spruceanum* and 16 *Philodendron* species), 2*n* = 34 (*T*. *mello-barretoanum* and 10 *Philodendron* species) and 2*n* = 36 (*T*. *corcovadense*, *T*. *lundii* and *T*. *saxicola*).

**Fig 1:**
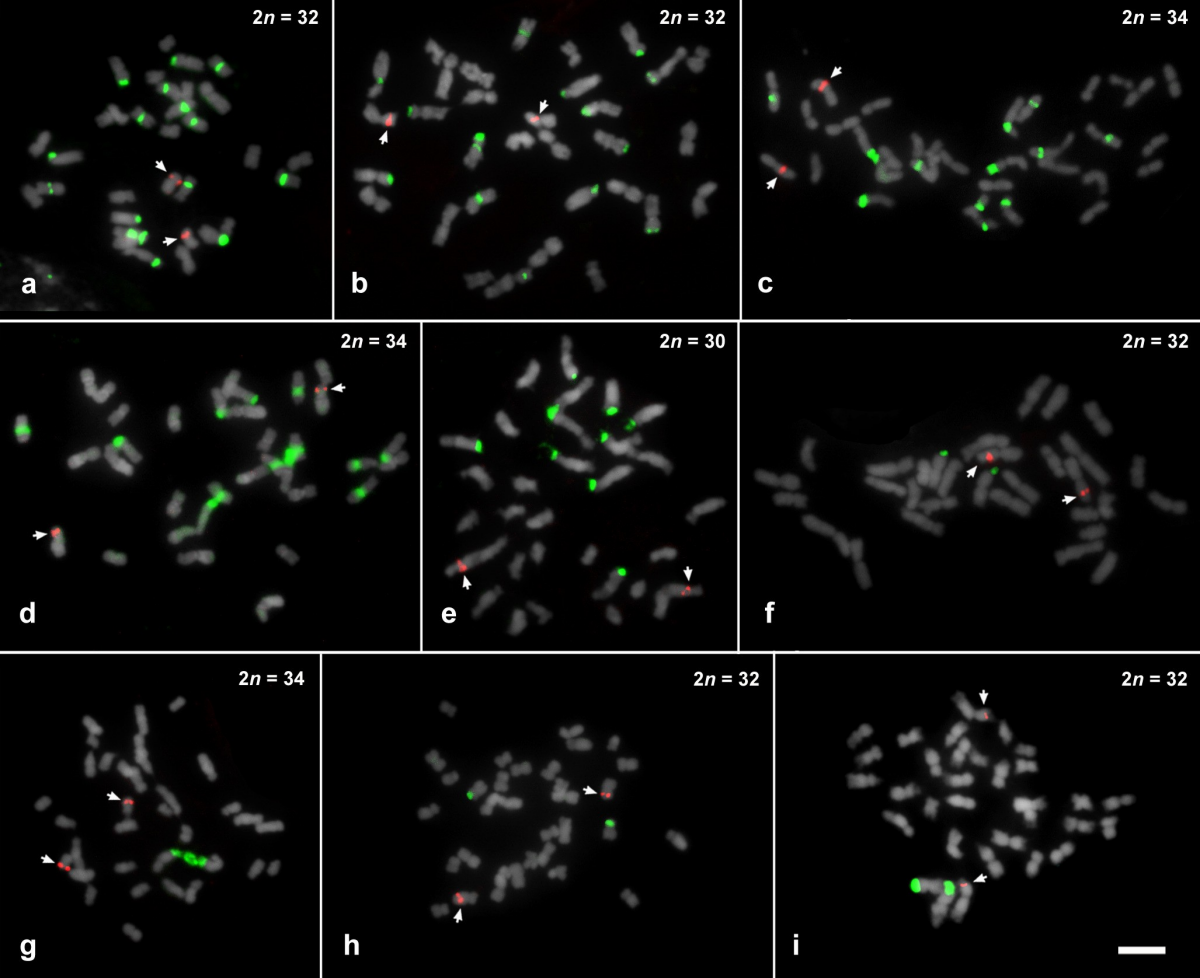
Fluorescent *in situ* hybridization of 35S (green) and 5S rDNA (red) on mitotic chromosomes of *Philodendron* species, counterstained with DAPI and pseudocolored in gray. (a) *Philodendron billietiae*; (b) *P*. *quinquenervium*; (c) *P*. *maximum*; (d) *P*. *uleanum*; (e) *P*. *giganteum*; (f) *P*. *distantilobum*; (g) *P*. *krugii*; (h) *P*. *nadruzianum*; (i) *P*. *fragrantissimum*. Bar in **i** represents 5 μm.

**Fig 2:**
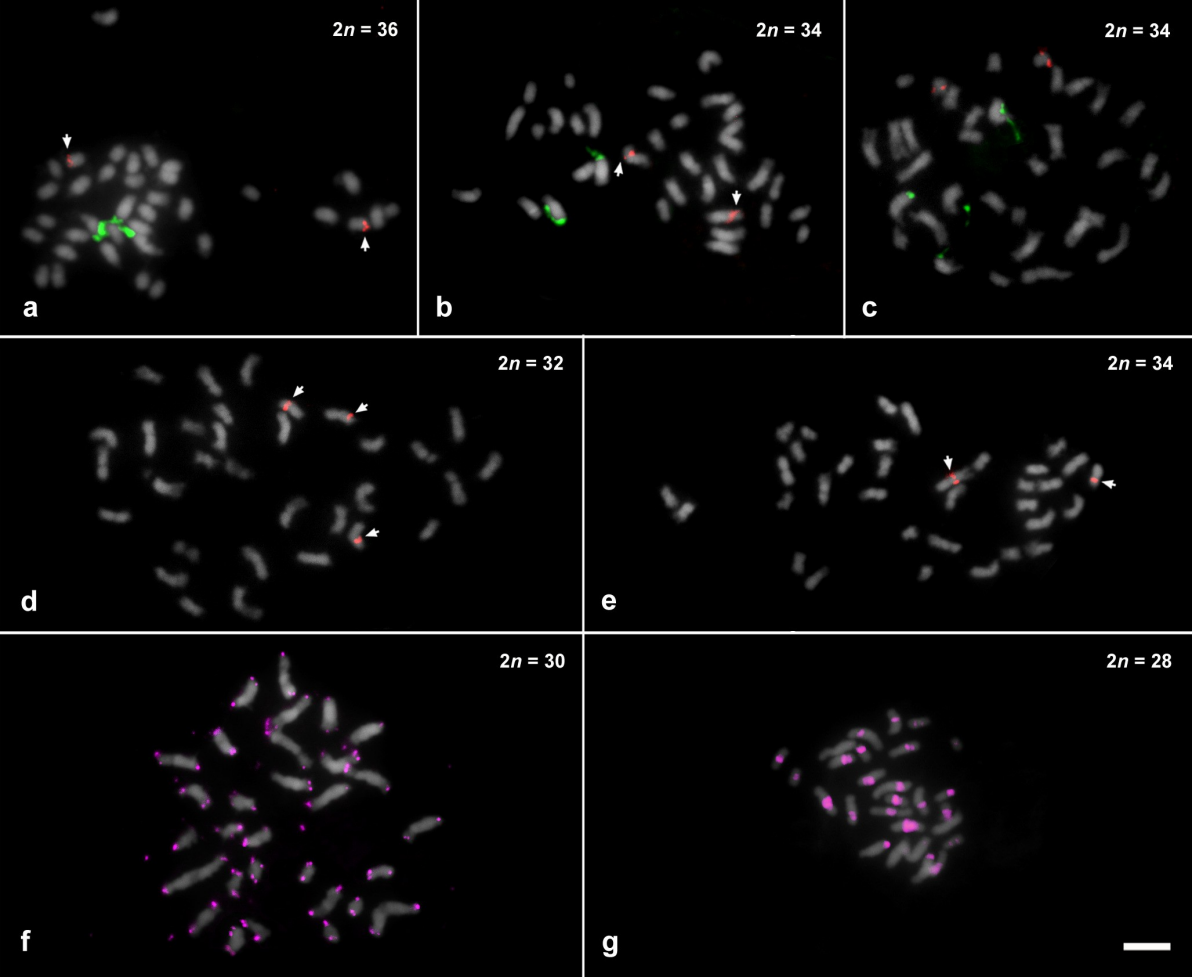
Distribution of repetitive sequences in species of *Thaumatophyllum* and *Philodendron* species, counterstained with DAPI and pseudocolored in gray (35S rDNA in green, 5S rDNA in red, and telomeric probe in pink). (a) *T*. *lundii*; (b) *T*. *mello-barretoanum*; (c) *P*. *angustilobum*; (d) *P*. *bipennifolium*; (e) *P*. *glaziovii*; (f) *P*. *giganteum*; (g) *P*. *callosum*. Bar in **g** represents 5 μm.

**Fig 3:**
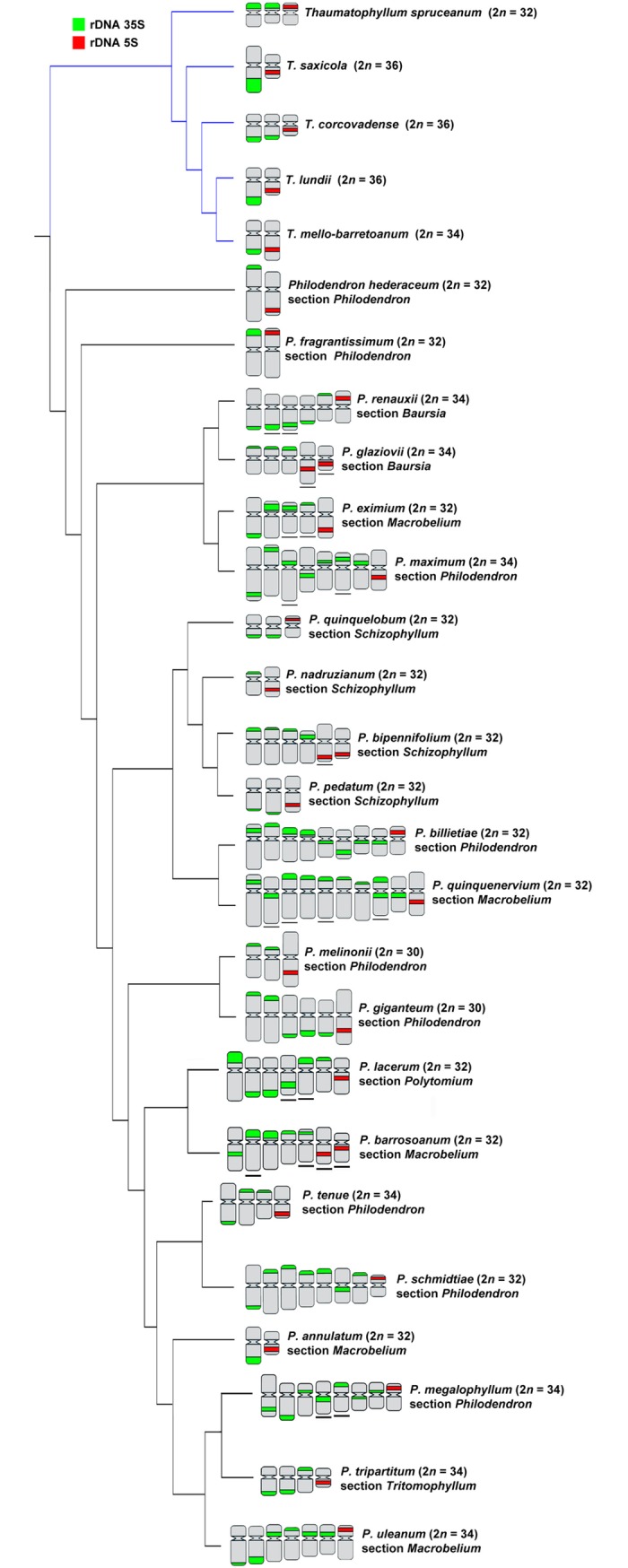
Chromosomal patterns for rDNA in 22 *Philodendron* and five *Thaumatophyllum* species plotted in a modified phylogeny based on Vasconcelos et al. [11]. Underlined chromosomes represent single chromosomes with heteromorphism, for which it was not possible to identify their respective homologs. Each non-underlined chromosome represents a pair of homologs.

**Fig 4:**
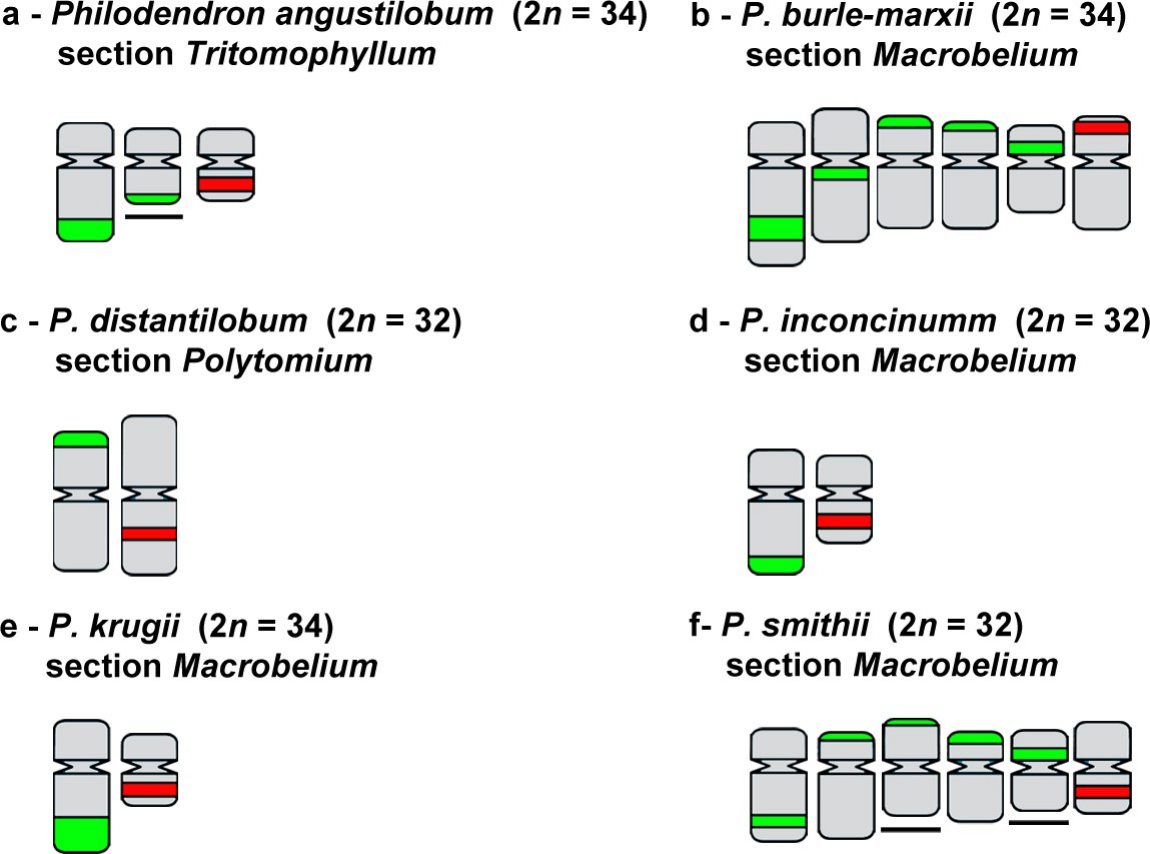
Chromosomes mapped with 35S (green) and 5S (red) rDNA probes in six species of *Philodendron* subgenus *Philodendron* not included on Vasconcelos et al. [11] phylogeny. Underlined chromosomes represent single chromosomes with heteromorphism, for which it was not possible to identify their respective homologs. Non-underlined chromosomes represent the pair of homologues.

FISH using 35S rDNA probe showed a wide variation in both number and location of sites along the chromosomes (see Table 1, Figs 1–4). For *Philodendron* species, *P*. *billietiae* (2*n* = 32, Fig 1A) showed the highest number of sites (16 sites), followed by *P*. *quinquenervium* (2*n* = 32, Fig 1B) with 15 sites; *P*. *schmidtiae* (2*n* = 32, Fig 3) with 14 sites; *P*. *maximum* (2*n* = 34, Fig 1C), *P*. *megalophyllum* (2*n* = 34, Fig 3) and *P*. *uleanum* (2*n* = 34, Fig 1D) with 12 sites; and *P*. *burle-marxii* (2*n* = 34, Fig 4), *P*. *giganteum* (2*n* = 30, Fig 1E) and *P*. *lacerum* (2*n* = 32, Fig 3) with 10 sites. In the other 19 species (with chromosome numbers ranging from 2*n* = 30 to 34), the number of sites varied from two to eight (Table 1, Fig 3). The lowest number of 35S rDNA sites was observed for seven species: *P*. *annulatum* (Fig 3), *P*. *distantilobum* (Fig 1F), *P*. *hederaceum* (Fig 3), *P*. *inconcinnum* (Fig 4), *P*. *krugii* (Fig 1G), *P*. *nadruzianum* (Fig 1H), and *P*. *fragrantissimum* (Fig 1I), which presented only two sites. For the five *Thaumatophyllum* species, two presented four 35S rDNA sites (*T*. *corcovadense* and *T*. *spruceanum*, Fig 3), and the other three had two sites (*T*. *lundii*, Fig 2A; *T*. *mello-barretoanum*, Fig 2B, and *T*. *saxicola*, Fig 3).

In *Philodendron*, the 35S rDNA sites were predominantly located in the terminal region of the short arm (21 out of the 29 species, Figs 1–4), although subterminal, interstitial or proximal sites were also observed. In *Thaumatophyllum*, the 35S rDNA sites were located only in the terminal region, predominantly in the long arm (four out of the five species, Figs 2 and 3). In addition, we did not observe a clear pattern of distribution of numbers of rDNA sites in the phylogenetic tree of *Philodendron* s.l. (Fig 3).

Heteromorphisms of number and distribution of 35S rDNA sites were identified in nine of the analyzed species. *Philodendron angustilobum*, for example, presented an odd number of 35S rDNA (three sites), hampering the identification of the homologous chromosomes (Fig 2C). Meanwhile, other species, as *P*. *eximium* and *P*. *smithii*, showed a chromosome pair bearing a 35S rDNA site in the terminal region of the short arm of one chromosome and in the proximal region of the short arm of the other one (Figs 3 and 4); *P*. *lacerum* and *P*. *megalophyllum*, presented sites located on opposite chromosome arms in supposed homologs (Fig 3); and *P*. *quinquenervium* was the only species with two 35S rDNA sites in the same chromosome (Fig 3).

In turn, for the 5S rDNA, most species showed two sites, except for *P*. *bipennifolium* (Fig 2D), which presented three sites. For *Philodendron*, we observed a high variation in the position of the 5S rDNA sites, although being most frequently observed in the interstitial position of the long arm (in 14 out of 29 analyzed species, Figs 1–4). For *Thaumatophyllum*, the 5S rDNA sites were located in the interstitial region predominantly in the long arm (four out of 5 species, Figs 2 and 3).

Both species *P*. *bipennifolium* and *P*. *glaziovii* stood out due to 5S rDNA heteromorphisms. The first had three 5S rDNA sites, being two located in the subterminal region of the long arm and one in the proximal region of the short arm (Fig 2D). In turn, *P*. *glaziovii*, had two 5S rDNA clusters, as most of the analyzed species, but the chromosomes bearing those sites were heteromorphic in size and morphology (Figs 2E and 3).

**Fig 5:**
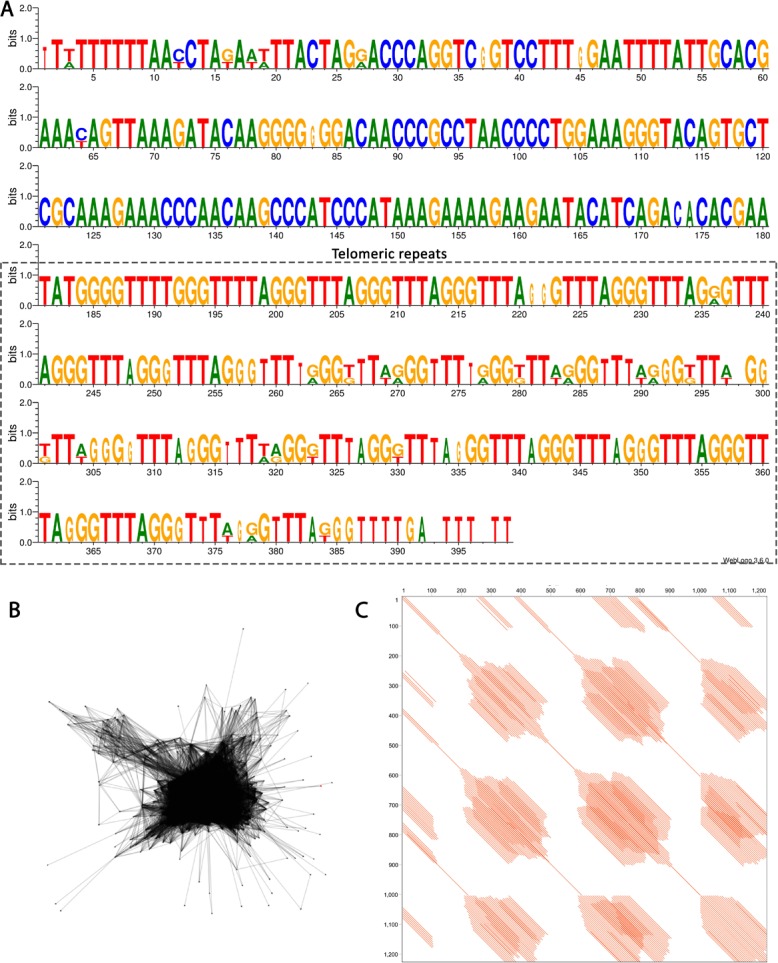
Features of the satellite DNA *Pc*Sat1 from *Philodendron callosum*. (a) Reconstructed monomer using WebLogo [63]. A cluster of conserved and degenerated plant telomeric motifs is present at the repeat unit and represents more than half of its full size (box). (b) Layout of cluster 228 obtained from Repeat Explorer output (Vasconcelos et al. unpublished data) and (c) Dotplot graph showing the internal organization of the sequence.

The telomeric DNA revealed no interstitial telomeric repeat (ITR) in *P*. *giganteum* (2*n* = 30), which exhibited only terminal marks in both extremities of all chromosomes (Fig 2F). In turn, *P*. *callosum* (2*n* = 28) presented no visible terminal hybridization signals, although exhibiting large pericentromeric marks in almost all chromosomes (Fig 2G). Analysis of the repetitive fraction of this species revealed the presence of one satellite DNA related to the telomeric repeat. The manual inspection of the contigs that composed the cluster CL228 allowed the identification of *Arabidopsis-*like telomeric motifs within the sequence, henceforth called *Pc*Sat1 satellite DNA. In all three monomers, the telomeric repeat was represented in variable amounts (unit 1 –U1, eight times; U2, 20 times; U3, nine times), making the monomer size slightly variable (Fig 5).

## Discussion

The present work is the first extensive cytogenetic study analyzing the localization of rDNA sites in chromosomes of *Philodendron* and *Thaumatophyllum* species, except for *P*. *hederaceum* [18]. Chromosome numbers are all in accordance with previously published data [9]. The results revealed an enormous karyotypic variability in relation to the distribution of the 35S rDNA in *Philodendron* s.l., opposed to a high stability in number and position of the 5S rDNA sites. These results are in consonance with the distribution pattern of rDNA sites in angiosperms and confirm the lower dispersion capacity of 5S rDNA proposed by Roa and Guerra [11, 12]. The presence of only one chromosome pair bearing 5S rDNA as found here seems to be conserved for the Araceae family (51 out of 54 analyzed species), even though only 12 out of 144 genera were analyzed up to date [17, 19].

Considering *P*. subg. *Philodendron* (with ca. 7,25% of its species analyzed), the amplification and distribution of 35S rDNA sites seem to occur frequently and independently along the different subgroups. The number of 35S rDNA sites (ranging from two to 16) varied significantly within and among the clades of the subg. *Philodendron*, with no clear phylogenetic pattern, considering the relationships presented by Vasconcelos et al. [21]. Also, there was not any correspondence of our data to the traditional subdivision of the subgenus in morphological sections.

*Thaumatophyllum* had fewer species analyzed, but better represented, with ca. 23.8% of the species of the genus. It showed a smaller variation in the number of rDNA sites (two to four sites), indicating a higher homogeneity among karyotypes. A similar homogeneity was observed in *Typhonium* (four 35S rDNA sites in eight out of 10 analyzed species), the aroid genus better studied cytogenetically so far [17].

In the present study, two of the 29 *Philodendron* species analyzed showed an odd number of rDNA sites, whereas for other 11 species, we observed heteromorphisms in site position or in the morphology of the chromosome bearing the rDNA. Similar polymorphisms have been previously described in Araceae and in other angiosperm families [19, 31, 32]. Considering that the rDNA sites are considered fragile, recombination hotspots may occur due to the highly repetitive nature of the locus, resulting in breaks followed by chromosomal rearrangements [33–35]. This could explain the interstitial position found for the 35S rDNA in some of our analyzed species, which is relatively uncommon in plant chromosomes [12]. Several hypotheses have been proposed for such polymorphisms, including the model of amplification, dispersion and deletion, the action of transposable elements, as well as non-homologous recombination, mainly related to the preferential terminal positions of the 35S rDNA sites on the chromosomes [34, 36–40]. The expansions and contractions of the repetitive DNA have frequently been associated to changes in the chromosome morphology, also resulting in changes in the position of rDNA sites [41], without necessarily changing the gene order [42].

Additionally, the diversity in the distribution of rDNA sites and the polymorphisms observed in *Philodendron* could be related to natural hybridizations that may have occurred throughout the evolutionary history of the group, although none of the analyzed species has a recognized hybrid origin. In *Citrus* L. (Rutaceae) and related genera, heteromorphism of chromosomal types is considered to be a reliable indicator of interspecific crosses, where variation in the number and location of CMA positive bands and rDNA sites is related to the fact that most species are apomictic hybrids [16, 31, 43–45]. Rapid changes in 35S rDNA loci in response to interspecific or intergeneric hybridization, when comparing to the progenitors, have also been reported in *Potamogeton* L. (Potamogetonaceae) [46], *Rosa* L. (Rosaceae) [47] and *Lolium* L. × *Festuca* L. [48, 49] hybrids.

Aiming to test the hypothesis of the existence of descending dysploidy events in *Philodendron* species generating lower chromosome numbers (2*n* = 28; 30) associated with centric or *end-to-end* fusion [19, 50, 51], we applied telomeric DNA probes to verify the possible presence of ITRs. Such internal telomeres were observed in dysploid series in *Nothoscordum* Kunth and *Ipheion* Raf., both belonging to the family Amaryllidaceae [52], as well as in *Typhonium* [17, 19]. However, the expected ITRs were not visible in *P*. *giganteum* (2*n* = 30), thus suggesting that the remnants of ITRs were lost along the karyotype evolution of this species by losing the chromosome extremities in translocation events, or by elimination or dispersion after insertion, as a result of high recombination rates in these regions, as suggested for *Phaseolus leptostachyus* Benth. [53]. Another plausible hypothesis would be that the ITRs are present in short arrays not detectable by FISH, as reported for tomato and for the bat *Carollia perspicillata* L. [54, 55].

In addition, FISH with the telomeric probe in *P*. *callosum* revealed several pericentromeric blocks related to a satellite DNA sequence. Telomeric repeats within satellite DNA arrays are not uncommon, being also reported in *Rumex induratus* Boiss & Reuter (Polygonaceae), *Jatropha curcas* L. (Euphorbiaceae), and in *Solanum lycopersicum* L. (tomato), *S*. *tuberosum* L. (potato) and *S*. *melongena* L. (eggplant) (Solanaceae) [54, 56–58]. AT-rich degenerated telomeric repeats were identified in the repeat St49 of *Solanum* L. species, indicated as an ancient sat-DNA derived from a telomeric-like sequence identified in the telomeres and centromeres of tomato and potato chromosomes, respectively [54, 58].

On the other hand, the absence of signals at chromosome termini of *P*. *callosum* may be due to the presence of small standard telomere arrays (TTTAGGG)_n_ not detected by FISH. However, the occurrence of a different telomere sequence in *P*. *callosum* chromosomes cannot be dismissed, as already described for several plant species [59–61]. This last scenario would imply in an interspecific variation within *Philodendron*, which was already reported for species of *Genlisea* A.St.-Hil. (Lentibulariaceae), a genus of carnivorous plants with a high variation level in the genome structures among species [59, 62].

## Conclusions

Our data revealed a substantial variation in the number and location of the 35S rDNA sites in *Philodendron* indicating a rapid karyotype evolution within *P*. subg. *Philodendron*. More homogeneous karyotypes were observed in species of the sister genus *Thaumatophyllum*. Variation was more prominent because no clear trend regarding the 35S rDNA sites was evident, neither considering the traditional infrageneric taxonomy nor considering the most recent molecular phylogenetic data. Also, the identification of heteromorphisms in the number and position of 35S rDNA sites suggests the occurrence of expansions and/or contractions of repetitive DNA in the genomes of some species, or chromosomal rearrangements, possibly associated with natural hybridization events. Furthermore, a better understanding of the importance of the repetitive DNA during the karyotypic evolution of *Philodendron* s.l. will be allowed by future analyses considering the evolution of chromosome numbers and genome sizes in a phylogenetic framework, besides a characterization of the composition of the repetitive DNA among species of the group, which are currently in development.
